# Comparison of Osteoblastic Differentiation of Human Periodontal Ligament Stem Cells through Application of Two β-tricalcium Phosphate Products: An *in vitro* Study

**DOI:** 10.30476/DENTJODS.2021.86700.1350

**Published:** 2022-06

**Authors:** Seyed Mohammad Hossein Mirkhani, Shirin Amini Sedeh, Vahid Esfahanian

**Affiliations:** 1 Postgraduate, Dept. of Periodontics, School of Dentistry, Isfahan (Khorasgan) Branch, Islamic Azad University, Isfahan, Iran; 2 Dept. of Periodontics, School of Dentistry, Isfahan (Khorasgan) Branch, Islamic Azad University, Isfahan, Iran

**Keywords:** Periodontal ligament, Alkaline phosphatase, Osteopontin, Bone Regeneration

## Abstract

**Statement of the Problem:**

Osteoblastic differentiation of periodontal ligament stem cells (PLSCs) is essential for alveolar bone regeneration.

**Purpose:**

The purpose of this study was to compare the potential of two β-tricalcium phosphate (βTCP) products to induce osteoblastic differentiation of human PLSCs.

**Materials and Method:**

In this *in vitro* study, human PLSCs were cultured in mediums supplemented with Guidor Easy-Graft [βTCP+polylactide-co-glycolide (PLCG)+n-methyl-2-pyrrolidone (NMP)] [Sunstar Company, Swiss] or Sorbone [βTCP] [Meta Company, South Korea] as two alloplasts experimental groups, mesenchymal cells differentiated into osteoblasts without alloplast as positive control group, and mesenchymal cells without osteoblastic induction as negative control group. Osteoblastic differentiation was evaluated using Alizarine Red staining and spectrophotometry to assay calcium deposits and real-time polymerase chain reaction to examine expression of alkaline phosphatase (ALP) and osteopontin (OPN) antigens on day 21. Data were analyzed by using SPSS 22 software and one-way ANOVA and Bonferoni tests (*p*< 0.05).

**Results:**

Spectrophotometry confirmed that calcium deposits were higher in Guidor Easy-Graft group compared to Sorbone group (*p*< 0.001) and higher in two experimental groups than controls (*p*< 0.05). According to real-time polymerase chain reaction, level of ALP expression was higher in Sorbone than Guidor and the levels of Guidor, positive control and negative control were equal; OPN levels of the positive control were more than the other groups. OPN levels of Sobone, Guidor and negative control were the same.

**Conclusion:**

These findings indicated that Guidor Easy-Graft and Sorbone enhanced differentiation of human PLSCs to osteoblasts, and could be employed as appropriate bone-graft materials.

## Introduction

In clinical dentistry, bone regeneration is used for treatment of alveolar bone resorption, ridge or socket preservation, preparation for dental implant placement and maxillary sinus floor augmentation [ 1 ]. Natural (autografts, allografts, and xenografts) or synthetic (alloplasts) materials implement bone grafting, as an approach to bone regeneration. Alloplasts including calcium phosphates (tricalcium phosphate, hydroxyapatite, and calcium polyphosphates), bioactive glasses, and calcium sulfate were introduced to overcome the natural material disadvantages. Disadvantages of autografts are related to limited intraoral access to the donor area as well as the amount of the tissue obtained [ 2 ]. Allografts and Xenografts may also lead to disease transmission or immune stimulation [ 3 ]. Alloplasts are synthetic, inorganic, and biocompatible materials used as bone substitutes and fillers for intraosseous defects, which can support bone regeneration during the osteoconduction process [ 4 ].

Two commercial beta-tricalcium phosphate (β-TCP) products include Guidor Easy-Graft and Sorbone. Guidor consists of a powder containing β-TCP particles, a thin polymer coating poly lactide-coglycolide (PLCG) and an activating liquid n-methyl-2-pyrrolidone (NMP) commercially named BioLinker. Sorbone contains pure beta-tricalcium phosphate [ 5 - 6 ]. Two previous studies have shown the capability to induce new bone formation by β-TCP exclusively [ 5 ] or in combination with PLCG [ 6 ]. PLCG can assist the formation of new bone due to calcium releasing capability [ 7 ] and also facilitates β-TCP application, increases its stiffness and stability, as well as improves its osteoconductivity and porosity as a scaffold [ 8 ].

Periodontal ligament stem cells (PLSCs) are subset of mesenchymal stem cells that can be easily extracted from periodontal tissue. These cells have a high ability to form new bone, cement, and periodontal ligament [ 9 ] and can differentiate into chondrocytes, osteoblasts, fibroblasts, and adipocytes under special conditions [ 10 - 11 ]. In particular, the ability of PLSCs to differentiate into osteoblasts has made them an appropriate candidate for the treatment of bone defects [ 12 ]. Osteoblastic differentiation of these cells could be confirmed by detection of differentiation markers including alkaline phosphatase (ALP) activity, mineralization capability, and expression of osteoblastic genes encoding ALP, osteopontin (OPN), osteocalcin, and bone sialoprotein [ 13 ]. Other methods including ALP staining [ 14 ], Alizarin Red staining for detection of tissue calcium [ 15 ] and polymerase chain reaction for determination of expression of different genes [ 16 ] are also performed to confirm osteoblastic differentiation. Stem cell differentiation is highly dependent on cell interaction with the physical, mechanical, and biological properties of the growth environment [ 8 ].

According to our search strategy, there was not any study on the effect of β-TCP with [(PLCG) + (NMP)] compound on osteoblastic differentiation of periodontal ligament stem cells. Therefore, the aim of this study was to evaluate the effects of β-TCP with or without [(PLCG) + (NMP)] compound on osteoblastic differentiation of human PLSCs.

## Materials and Method

Human PLSCs (IBRC cell code C11326) were obtained from the cell bank of Iranian Biological Resource Center.

Human PLSCs stored in liquid nitrogen at -196 °C were melted at 37°C. The cell suspension was transferred into a tube containing complete cell culture medium, Dulbecco's Modified Eagle Medium, with 5% Fetal Bovine Serum. After centrifugation at 200 g for 5min, 1ml of fresh medium (10% Fetal Bovine Serum + Dulbecco's Modified Eagle Medium-F12) was added to the superficial layer separated from centrifuged cell suspension. The cells were grown in complete culture medium containing Fetal Bovine Serum to obtain a sufficient number of cells. The prepared suspension was cultured in four plates each one containing 24 wells aka (96 wells) and one plate containing 24 wells, and then were incubated.

When the cell density in the plates reached more than 70%, cell passage was performed to provide sufficient nutrients and remove waste products.

The cells were cultured in four groups including Guidor group, Sorbone group, positive control group (mesenchymal cells differentiated into osteoblasts without alloplast), and negative control group (undifferentiated mesenchymal cells).

The plates containing 24 wells were used to evaluate cells by Alizarin Red test and the plates containing 96 wells were used to examine cells by polymerase chain reaction. The experiments were repeated four times for each group. 

To evaluate the differentiation into osteoblasts, PLSCs of the fourth passage were cultured in Dulbecco's Modified Eagle Medium (medium containing 10% bovine serum, dexamethasone, 0.2mM ascorbic acid, and 10mM beta-glycerophosphate) to induce bone differentiation. The cells were transferred to culture flasks at a concentration of one million cells per milliliter and they were incubated under normal cell culture conditions. After 48 hours, the flask culture medium was changed and then the cell culture medium was changed every three days. When the cell density reached 80%, cell passage took place. As a negative control group, the cells were cultured in Dulbecco's Modified Eagle Medium containing 10% bovine serum. The cells were then incubated at 37°C and 5% CO2 for 21 days. Their medium was changed every two days. At the end of this period, the occurrence of cell differentiation was explored by Alizarin Red staining. Thereby, the cell monolayer was washed with phosphate buffered saline and fixed by pure methanol for 10 min, then stained with 2% Alizarin Red dye in 25% ammonia water for 2 min. Finally, the cells were washed with distilled water, dried, and then examined by a microscope.

Using the "ELISA Reader" spectrophotometer, the quantity of 405 nm light absorption was measured as a marker for the amount of calcium deposits. The expression of ALP and OPN genes, which indicate differentiation into osteoblast cells, was measured by polymerase chain reaction technique in all groups. In our study, osteoblastic differentiation of PLSCs was assessed by Alizarin Red staining and polymerase chain reaction on day 21, the time that required for differentiation of these cells [ 17 ]. Data were analyzed by SPSS 22 software using one-way ANOVA and Bonferoni tests (*p*< 0.05).

## Results

**Figure 1:**
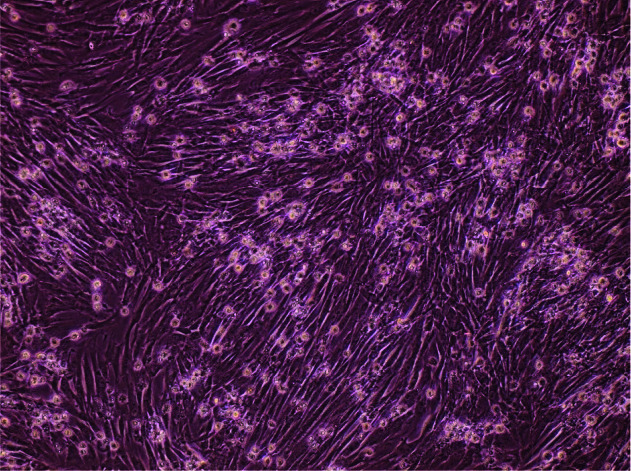
Microscopic image of cells in the negative control group after Alizarin Red staining (10x magnification)

**Figure 2:**
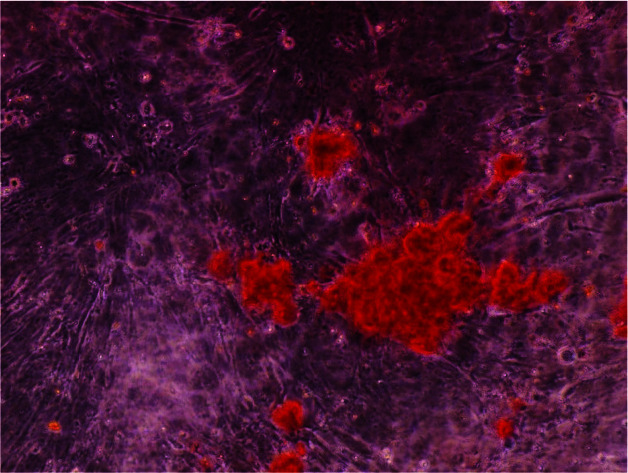
Microscopic image of cells in the positive control group after Alizarin Red staining (10x magnification)

**Figure 3:**
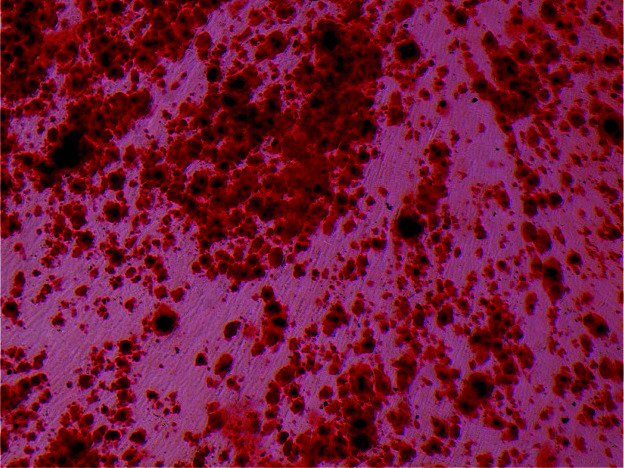
Microscopic image of cells in Sorbone group after Alizarin Red staining (10x magnification)

**Figure 4:**
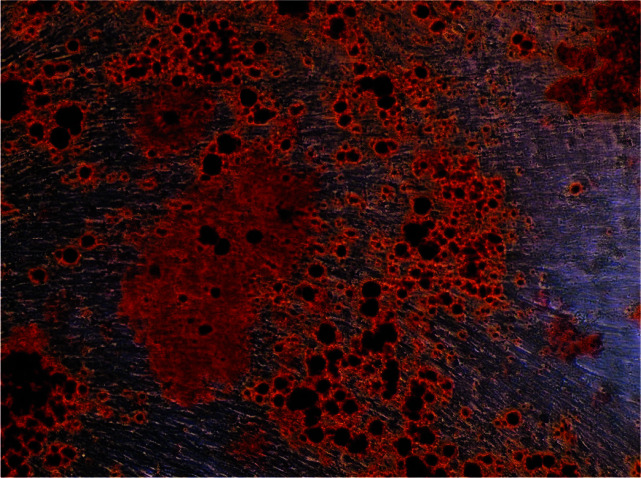
Microscopic image of cells in Guidor group after Alizarin Red staining (10x magnification)

Microscopic evaluation of Alizarin-stained samples indicated that the negative control samples had no staining (absence of differentiation; Figure 1) and the positive control samples had partial staining (relative differentiation; Figure 2). Samples in the Sorbone group (Figure 3) and the Guidor group (Figure 4) showed the highest staining (high differentiation). Spectrophotome try confirmed that calcium deposits were higher in Guidor group compared to Sorbone group (*p*< 0.001) and higher in two experimental groups than controls (*p*< 0.05) (Table 1).

**Table 1:** Mean light absorption in samples stained with Alizarin Red after 21 days

Group	Mean ± SD	*p* Value*
Guidor	0.6349 ± 0.0036^a^	< 0.001
Sorbone	0.5767 ± 0.0038^b^	
Positive control	0.1037 ± 0.0022^c^	
Negative control	0.0109 ± 0.0000^d^	

* One-way ANOVA, Groups with significant differences in gene expression were marked with different superscript letters (Bonferroni test,*p*< 0.05)

The mean expression of ALP gene in Sorbone group was significantly higher than Guidor group (*p*= 0.005), negative and positive controls (*p*= 0.001). There was no significant difference between Guidor, negative control, and positive control groups (*p*> 0.05). The mean expression of OPN gene in the positive control group was significantly higher than Guidor (*p*= 0.010), Sorbone (*p*= 0.018) and negative control (*p*= 0.001) groups. There was no significant difference between Sorbone, Guidor, and negative control groups (*p*> 0.05) (Table 2).

**Table 2:** Mean expression of alkaline phosphatase and osteopontin genes after 21 days

Gene	Group	Mean±SDp	*p* Value*
Alkaline phosphatase	Guidor	9.190±4.659^a^	< 0.001
	Sorbone	36.958±16.918^b^	
	Positive control	3.191±1.764^a^	
	Negative control	1.000±0.000^a^	
Osteopontin	Guidor	2.767±0.305^a^	0.001
	Sorbone	3.258±1.390^a^	
	Positive control	9.002±4.138^b^	
	Negative control	1.000±0.000^a^	

* One-way ANOVA, For each gene, groups with significant differences in gene expression were marked with different superscript letters (Bonferroni test, *p*<0.05)

## Discussion

Alloplasts have been known as a practical option to treat bone defects due to periodontal diseases. β-TCP materials are very common practical alloplasts. β-TCP has a crystalline structure and its porosity provides desirable biological properties such as osteoconduction and resorption. It also causes the formation of new bone within 3 to 6 months by releasing calcium and phosphate ions [ 18 ]. The effects of β-TCP in pure form or in combination with other substances on the differentiation of PLSCs into other cell lines have been previously studied [ 19 - 20 ].

In the present study, human PLSCs were used because of their several beneficial properties. These stem cells have the ability to differentiate into various cells such as osteoblasts, odontoblasts, adipocytes, and neuron-like cells. They are also superior to other dental stem cells in terms of proliferation and specific transcription factors and simultaneous with periodontal tissue regeneration, they can cause the formation of cementum-like and periodontal ligament-like structures. Finally, they decrease inflammatory reactions and immune responses [ 21 ].

In our study, Alizarin Red staining/ spectrophotometry and expression of ALP and OPN genes were used to evaluate osteoblastic differentiation. Alizarin Red staining/ spectrophotometry demonstrate the deposition of minerals associated with osteoblast differentiation; hence, identifies the osteoblastic phenotype. In addition, the expression of ALP and OPN genes are used to identify genes associated with this phenotype [ 22 ].

In this study, four groups including two experimental groups (Guidor and Sorbone groups), one positive control group, and one negative control group were evaluated. Evaluation of osteoblast differentiation by spectrophotometry showed that calcium deposition was negligible in the negative control group. Therefore, the increase in calcium deposition in the other three groups can be attributed to its production by osteoblasts differentiated from the stem cells.

Calcium deposition was significantly higher in Guidor group and Sorbone group than positive control group. This finding indicates that these two materials had a significant effect on increasing osteoblastic differentiation of human PLSCs. The main component of Guidor and Sorbone is β-TCP. Similar to our findings, two previous studies [ 23 - 24 ] have shown that calcium phosphate could increase bone formation, osteoblast proliferation, and activity. In a clinical study, Naineni *et al.* [ 25 ] found that the usage of β-TCP-containing materials in patients with alveolar bone defects inhibited alveolar crest degeneration and enhanced bone formation. Amalakara *et al.* [ 26 ] reported that β-TCP alone or in combination with an immunosuppressive agent (cyclosporine A) improved alveolar bone growth and caused filling of bone defects. According to a clinical trial by Cochran *et al.* [ 27 ], periodontal bone defects were treated by β-TCP/rh-FGF-2. Maroo and Murthy [ 28 ] evaluated patients with periodontal intraosseous defects and reported the efficacy of β-TCP/rhPDGF combination in filling bone defects and increasing alveolar margin height.

Calcium deposition was significantly higher in Guidor group compared to Sorbone group. Although the main component of both products is β-TCP, there are differences in composition and structure between two products. Guidor contains PLCG that is activating by liquid (NMP). It has been shown that PLCG supports the formation of new bone by releasing calcium [ 7 ]. Therefore, the higher calcium deposition in Guidor group than Sorbone group can be attributed to the presence of PLCG in Guidor.

The mean expression of ALP gene in Sorbone group was significantly higher than the positive control. This finding indicates that the expression of ALP gene as a marker of osteoblastic differentiation increased under the influence of Sorbone. A study on animal cells showed that incubation of bone marrow stromal cells in the presence of β-TCP significantly increased ALP production [ 5 ]. McCafferty *et al.* [ 29 ] observed that the addition of calcium phosphate coating on titanium surfaces improved the osteogenic differentiation of mesenchymal stem cells cultured on these surfaces. According to An *et al.* study [ 19 ], culturing human PLSCs in the presence of β-TCP, increased the ALP activity.

However, ALP gene expression in Guidor group was lower than Sorbone group and similar to positive and negative controls. The difference between Guidor and Sorbone groups may be explained by the inhibitory effect of PLCG and NMP in Guidor product. On the other hand, because ALP gene expression occurs at the beginning of differentiation, it may be present in any group, and as a result, ALP gene expression is the same in the negative and positive control groups. Kouhestani *et al.* [ 30 ], examined the effect of hydroxyapatite/β-TCP granules on bone differentiation of dental pulp stem cells, and found no significant difference in ALP activity between the hydroxyapatite/β-TCP group and the control group, which is similar to findings of the Guidor group in our study. Although there is no statistically significant difference between positive control group and negative control group in the mean expression of ALP gene, but quantitatively, the mean expression of this gene in the positive control group is higher than the negative control group (Table 2).

It is possible that in other experimental conditions such as increasing the sample size and so on, a statistically significant difference was achieved between the positive control group and the negative control group.

The mean expression of OPN gene in the positive control group was significantly higher than Guidor and Sorbone groups. There was no significant difference in the expression of OPN gene between Sorbone, Guidor, and negative control groups. Based on these findings, it appears that the expression of some genes is not induced in differentiated osteoblasts. Similar to this finding, Beck et al. [ 31 ] found that expression of osteoblast-specific genes was significantly suppressed in osteoblasts differentiated from some stem cell lines. The presence of alloplastic material may also have an inhibitory effect on the expression of some genes and the better expression of OPN gene in the positive control group than the other three groups can be attributed to it. In addition, unlike the expression of the ALP gene that occurs during the early stages of differentiation, the expression of the OPN gene is related to the higher stages of cell differentiation. Therefore, the lack of expression of this gene can be attributed to the 21-day evaluation period in the present study, which may have been insufficient time for the expression of this gene. 

Xia *et al.* [ 20 ],investigated the osteogenic differentiation of human PLSCs based on the expression of ALP and OPN genes and reported that the expression of the genes was lower in β-TCP group than acermanite group. In addition, An *et al.* [ 19 ], observed that during osteogenic differentiation of human PLSCs, the expression of osteocalcin gene in the presence of β-TCP was significantly lower than the demineralized bone matrix. However, Miron *et al.* [ 23 ] compared the effects of autograft, allograft, xenograft, and calcium phosphate on osteoblastic differentiation of bone marrow stem cells and reported that the osteocalcin gene was expressed only in the presence of calcium phosphate and autograft. It seems that the effects of β-TCP on osteoblastic differentiation of stem cells and the expression of specific genes are affected by the type of stem cells, their preparation methods and culture medium formula, and the storage conditions. It can also be affected by presence of other compounds with β-TCP, as well as the shape and size of β-TCP particles.

As a limitation, this study was performed in vitro and generalization of results to clinical conditions should be performed with caution.

## Conclusion

It could be concluded that two commercial β-TCP products with or without combination [(PLCG) + (NMP)] might assist the differentiation of human PLSCs into osteoblasts; thus, improve the bone regeneration. However, β-TCP in combination with [(PLCG) + (NMP)] showed some degree of inhibitory effect on ALP gene expression but it could play a role in improving calcification. Guidor Easy-Graft and Sorbone did not differ significantly in terms of OPN gene expression.

## Conflict of Interest

 The authors declare that they have no conflict of interest.
